# Role of NK Cell Subsets in Organ-Specific Murine Melanoma Metastasis

**DOI:** 10.1371/journal.pone.0065599

**Published:** 2013-06-11

**Authors:** Zuhair K. Ballas, Claire M. Buchta, Timothy R. Rosean, Jonathan W. Heusel, Michael R. Shey

**Affiliations:** 1 Iowa City VA Medical Center and the Department of Internal Medicine, University of Iowa Carver College of Medicine, Iowa City, Iowa, United States of America; 2 Interdisciplinary Graduate Program in Immunology, University of Iowa Carver College of Medicine, Iowa City, Iowa, United States of America; 3 Department of Pathology, University of Iowa Carver College of Medicine, Iowa City, Iowa, United States of America; Vanderbilt University Medical Center, United States of America

## Abstract

Tumor metastasis plays a major role in the morbidity and mortality of cancer patients. Among solid tumors that undergo metastasis, there is often a predilection to metastasize to a particular organ with, for example, prostate cancer preferentially metastasizing to bones and colon cancer preferentially metastasizing to the liver. Although many factors are thought to be important in establishing permissiveness for metastasis, the reasons for organ-specific predilection of each tumor are not understood. Using a B16 murine melanoma model, we tested the hypothesis that organ-specific NK cell subsets play a critical role in organ-specific metastasis of this tumor. Melanoma cells, given intravenously, readily colonized the lungs but not the liver. NK cell depletion (either iatrogenically or by using genetically targeted mice) resulted in substantial hepatic metastasis. Analysis of NK cell subsets, defined by the differential expression of a combination of CD27 and CD11b, indicated a significant difference in the distribution of NK cell subsets in the lung and liver with the mature subset being dominant in the lung and the immature subset being dominant in the liver. Several experimental approaches, including adoptive transfer, clearly indicated that the immature hepatic NK cell subset, CD27+ CD11b–, was protective against liver metastasis; this subset mediated its protection by a perforin-dependent cytotoxic mechanism. In contrast, the more mature NK cell subsets were more efficient at reducing pulmonary tumor load. These data indicate that organ-specific immune responses may play a pivotal role in determining the permissiveness of a given organ for the establishment of a metastatic niche.

## Introduction

Tumor metastasis plays a major role in the morbidity and mortality of cancer patients. It is estimated that at least 90% of cancer mortality is due to metastatic lesions rather than the primary tumor itself. It is well established that, among solid tumors that undergo metastasis, there is often a predilection to metastasize to particular organs. For example, prostate cancer metastasizes primarily to bone while colon cancer implants in the liver. The reasons for organ-specific metastasis, the so-called “metastatic niche”, are not well defined. There are several possible explanations for an organ dominance, some of which may be tumor specific and others that may be organ-specific, including the expression of adhesion molecules, vascular growth factors, extracellular matrix composition, and coagulation factors. From an immunological point of view, various lymphocyte subsets have different requirements for trafficking to various organs including primary and secondary lymphoid tissues as well as non-lymphoid organs. It is possible that there are organ-specific immune responses that might be able to eliminate a metastatic clone in one organ but not another [1]–[5].

We had previously described that CpG oligodeoxynucleotides (ODN), given intraperitoneally (i.p.), were able to prevent the establishment of B16 melanoma and to prolong the survival of mice with established B16 melanoma. We also showed that natural killer (NK) cells were necessary and sufficient for this effect [6]. We did not examine the role of specific NK cell subsets in such a response. It is now well established that NK cell are comprised of various subsets that can be defined both functionally and by a combination of surface markers. Functionally, NK cells can be divided in two major subsets: those that secrete cytokines (IFN-γ in particular) and those that are cytotoxic. Various combinations of surface markers have been examined but there is no consensus as to which combination best characterizes these functional subsets [7]–[11]. One of the more useful surface marker examinations employed the combination of CD27 and CD11b. This combination appears to define a continuum of NK cell maturation with the most immature NK cells being negative for both markers followed by sequential acquisition of CD27 and CD11b followed by down modulation of CD27. Thus, CD27^−^ CD11b^−^ cells are the most immature while the CD27^−^CD11b^+^ cells are the most mature [7], [8], [10], [12], [13].

In our experiments with B16 melanoma, we found that when the tumor was given intravenously (i.v.), it colonized the lungs but not the liver or the peritoneum. Since we have previously shown that NK cells are necessary and sufficient (in conjunction with CpG ODN administered in vivo) to prevent establishment of the tumor, and to prolong survival in mice with established tumor, the question was whether organ-specific NK cell subsets might be responsible for this dichotomy between the lungs and liver [6].

The experiments in this report were undertaken to test the hypothesis that organ-specific NK cell subsets will determine whether that organ is permissive for establishment of B16 melanoma.

Our results indicate that a CD27^hi^ CD11b^lo^ hepatic NK subset is indeed important in preventing hepatic but not pulmonary metastasis. Moreover, we show that perforin-dependent mechanisms (i.e. cytotoxicity) are pivotal in this protection while IFN-γ and IL-12 are not necessary. Our studies with adoptive transfer of NK cell subsets also indicate that there is an organ-specific hierarchy of protection exerted by various NK cell subsets with the mature subsets being more protective in the lung while the immature subsets were more protective in the liver.

## Results

### Intravenous Injection of B16 Melanoma Results in Lung Colonization but Spares the Liver

**Figure 1:**
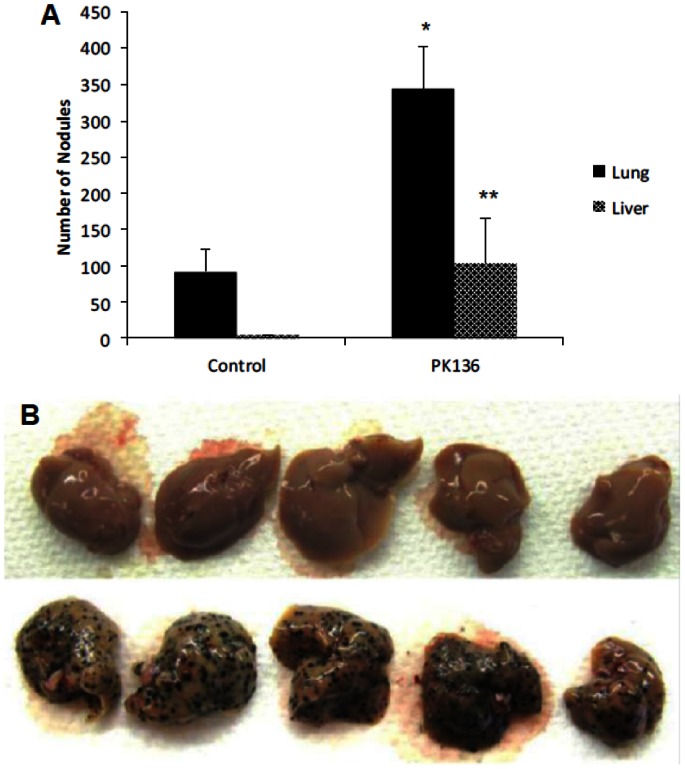
NK depletion results in establishment of liver tumor nodules and increased pulmonary tumor load. B6 mice were given control Ig or PK136 on day −3 and day 0 (i.p.) followed by i.v. injection of B16 tumor on day 0. Mice were euthanized on Day 14 and tumor nodules counted; there were five mice per group. The increase in tumor nodules in the lung was significant at p = 0.019 (indicated by *). The increase in liver tumor nodules was significant at p = 0.005 (indicated by **). The design of the experiment was such that all mice groups were processed simultaneously. A visual depiction of a similar experiment is in (B) where the bottom row shows livers from NK-depleted mice.

B16 melanoma was injected intravenously into wild type B6 mice at day 0. Mice were euthanized on days 12–14 and examined for melanoma lesions in various organs. We found significant colonization in the lungs but none in the liver (Fig. 1A). When B6 mice were depleted of NK cells by the injection of mAb PK136 mAb (anti-NK1.1) on day −3 and day 0, we found a dramatic acquisition of hepatic tumor (Figure 1A), which was easily visualized. The top row in Figure 1B indicates individual livers from wild type (WT) mice that received B16 and that were treated with control antibody on day −3 and day 0. The bottom row in Figure 1B indicates individual liver from WT mice whose NK cells were depleted. As shown in Figure 1A, the number of lung melanoma nodules also increased upon depletion of NK cells.

**Figure 2:**
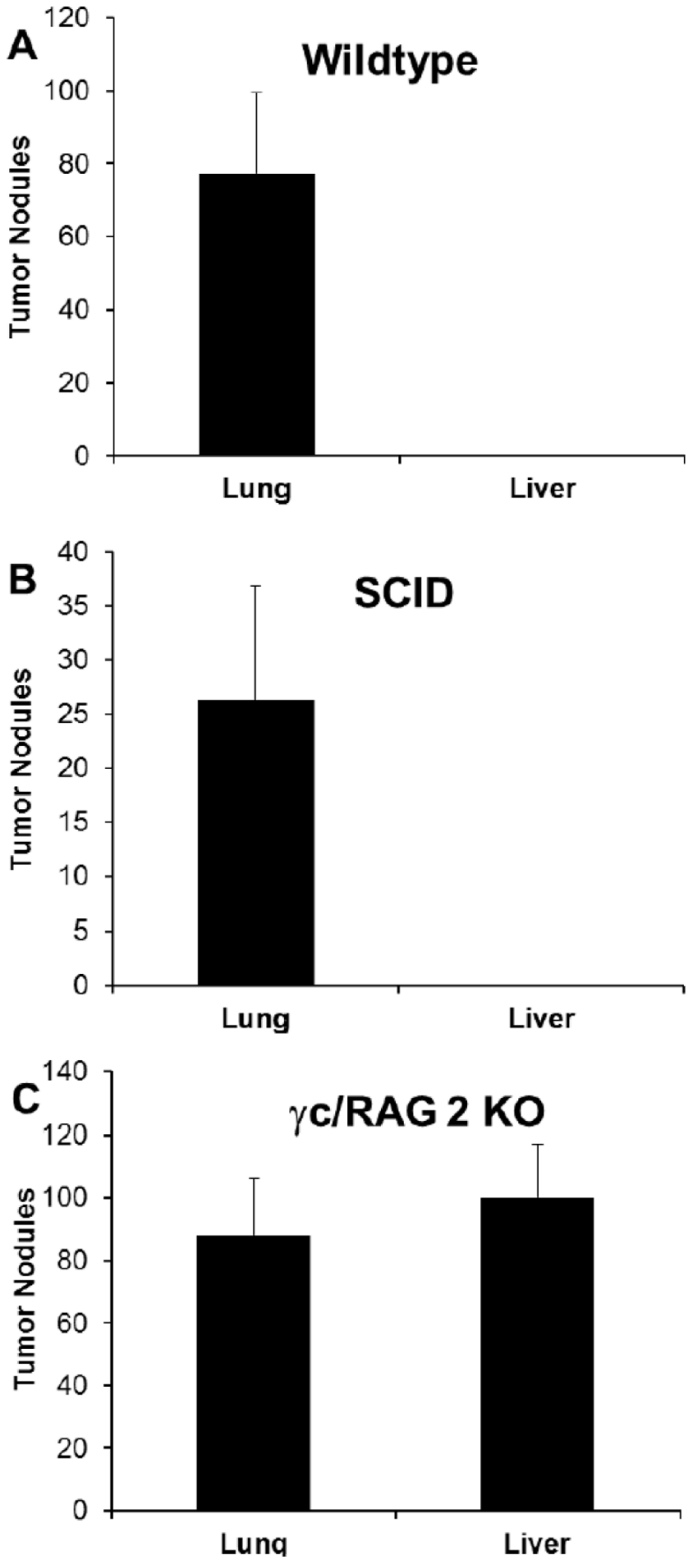
Intravenous inoculation with B16F1 melanoma results in the establishment of liver tumor nodules in NK cell deficient mice. 2×10^5^ melanoma cells were injected i.v. into (A) wild-type, (B) SCID or (C) γc/RAG 2 KO mice (all on a C57BL/6 background). Twelve days later, lungs and livers were excised and tumors enumerated. There were five mice per group.

PK136 recognizes a polymorphic epitope (termed NK1.1) of NKRP-1C, which is present both on NK and NKT cells [14], [15] [16]. It appeared possible that it is the NKT rather than the NK cells which are important in preventing liver metastasis. This was tested directly by treating SCID mice (which have NK but not NKT cells) with control Ig or PK136. Similar to WT (Fig. 2A), SCID mice given B16 melanoma i.v. develop only pulmonary tumor with no liver involvement (Fig. 2B). PK136-mediated depletion of NK cells in SCID mice resulted in an increase in the number of tumor nodules in the lung and a significant colonization of the liver as well (data not shown). To further confirm the importance of hepatic NK cells, B16 melanoma was given intravenously to γc RAG-2 knockout mice (which lack T, B and NK cells). These mice displayed both pulmonary and hepatic implantation (Fig. 2C).

These results suggested that hepatic NK cells, under normal circumstances, prevent the colonization of the liver by B16 melanoma. Since NK depletion (iatrogenically or using genetically targeted, NK cell-deficient mice) resulted in increased pulmonary colonization as well, one could hypothesize that pulmonary NK cells exert some (but not total) protection against lung metastasis while hepatic NK cells exercise total protection.

### Role of NK Cytotoxicity vs. Cytokine Secretion in Liver Protection

**Figure 3:**
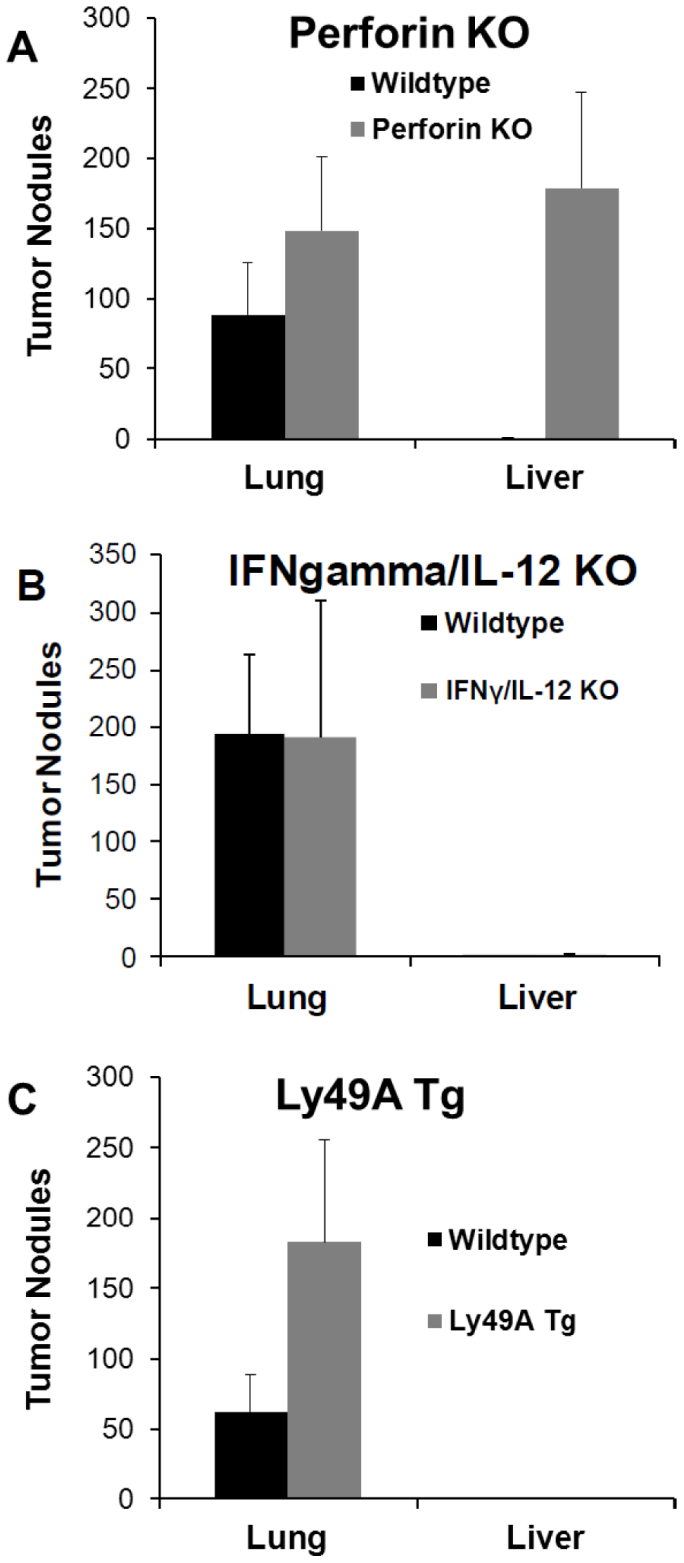
Cytolytic machinery but not IFN-gamma mediates protection from liver tumor colonization. (A) Perforin KO mice or wild-type B6 were injected with 3×10^5^ B16F1 i.v. and euthanized 12 days later. The difference in tumor nodules in lungs was not significant while the difference in liver nodules was highly significant (p = 0.004). (B) IFN-γ/IL-12 KO or wild-type B6 mice were injected with B16F1 i.v. and euthanized 12 days later. The differences were not significant (C) Ly49A Tg or wild-type littermate control mice were injected with B16F1 i.v. and euthanized 12 days later. The number of pulmonary tumor nodules was significantly increased in Ly49A Tg mice as compared to WT (p = 0.018). The design of the experiments was such that all groups in each panel were examined simultaneously.

NK cells can be divided in two broad subsets defined by their predominant effector function: cytotoxicity or cytokine secretion (primarily IFN-γ). In order to determine which NK cell effector mediates protection against hepatic B16 colonization, we examined perforin knockout mice (which are deficient in granule-mediated cytotoxicity) as well as IFN-γ/IL-12 double knockout mice. As shown in Figure 3, it appeared that cytotoxic mechanisms rather than IFN-γ/IL-12 are important in hepatic protection since perforin −/− mice developed liver tumors while the IFN-γ/IL-12−/− mice did not.

In order to explore further the potential NK cell subset responsible, we examined Ly49A transgenic (LY49At) mice. These mice have a deficiency of mature NK cells in the periphery since NK cell development is blocked at an immature stage secondary to a positional effect of the integrated transgene [17], [18]. Interestingly, we found that these mice had more extensive pulmonary metastasis (as expected) but they had no hepatic involvement (Fig. 3C). This suggested that the liver might contain an immature NK cell subset previously uncharacterized in this strain, which may be involved in controlling local metastasis.

**Figure 4:**
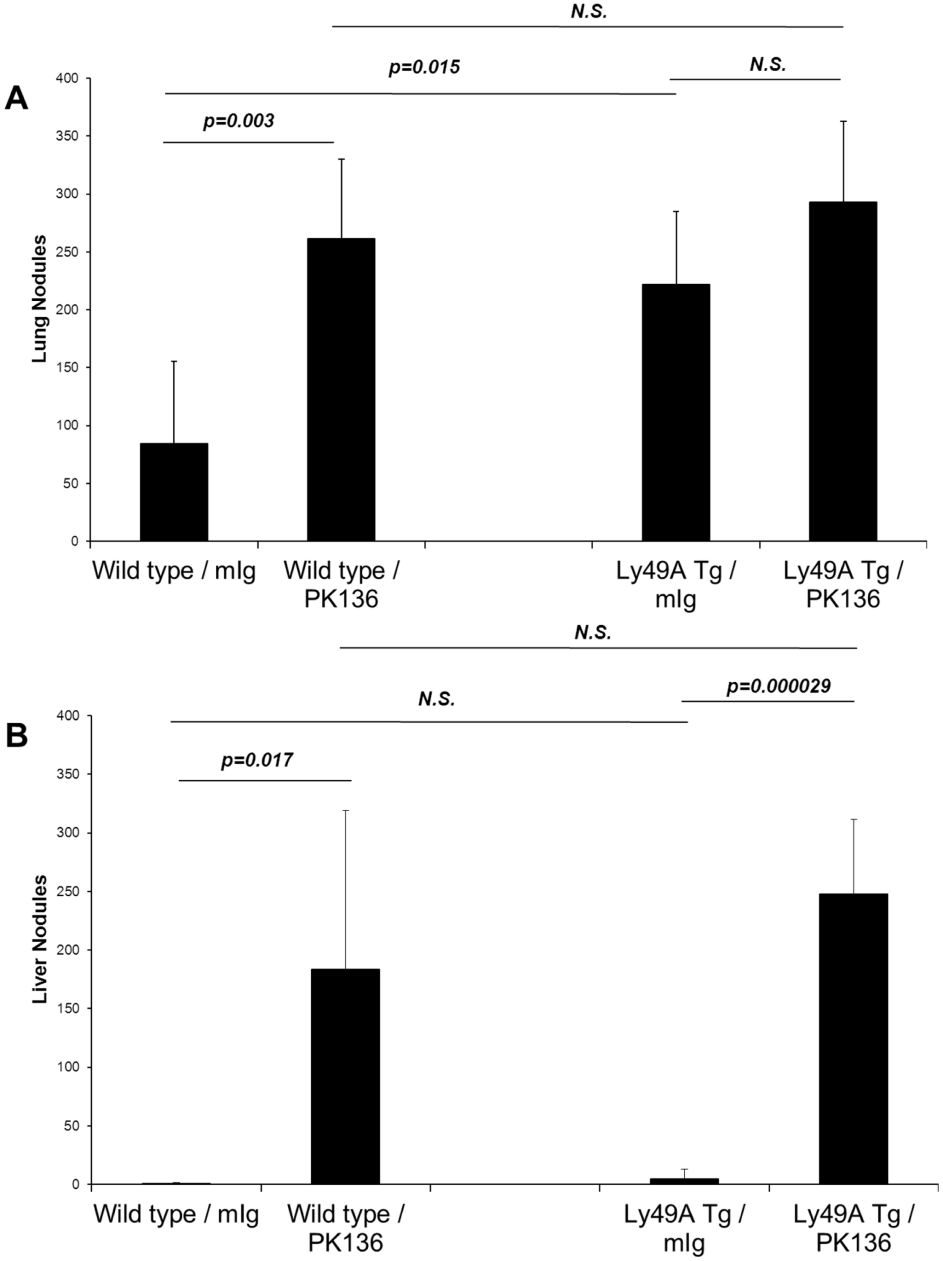
Arrested maturation of NK cells exacerbates lung tumor engraftment, but has minimal impact on liver tumor load. Wild type or Ly49A transgenic mice were treated with control mouse immunoglobulin, or depleted of NK cells using mAb PK136. 3×10^5^ B16F1 melanoma cells were injected i.v., and mice were euthanized 14 days later for enumeration of tumor burden. Ly49A Tg mice develop more pulmonary tumor nodules than wild type littermates. Elimination of NK cells does not significantly increase the number of tumor nodules in the lung. In contrast, Ly49A Tg mice develop a similar number of liver tumor nodules as wild type controls, indicating that the immature hepatic NK cells confer substantial protection against tumor establishment in this organ.

Of note is that Ly49At mice consistently displayed a much larger tumor load as compared to WT mice. One explanation for such a finding is that the mature NK cell subset (which is deficient in Ly49At mice) is the subset that can exert a protective effect in the lung. If this were the case, one would expect that depleting all NK cells in Ly49At mice (by injecting PK136) would have no effect on the pulmonary tumor load. As shown in Figure 4A, Ly49At mice again had a larger pulmonary tumor load than the WT mice. NK depletion, while increasing the pulmonary tumor load in WT mice, did not do so in the Ly49At mice. Interestingly, however, NK depletion in the Ly49At mice did result in the establishment of hepatic tumor in both strains (Figure 4B). There was no significant difference in the hepatic tumor load between the WT and Ly49At mice suggesting the NK cell subset important in the liver is probably an early subset along the maturation pathway which is before the maturation block exerted by the Ly49A insertion.

### Organ-specific NK Subset Distribution

Phenotypically, murine NK cell subsets can be subdivided by any number of surface markers. Although there is no consensus as to the optimal marker combination that can define NK cell maturation stages, there is significant support in the literature for the use of the co-expression of CD27 and CD11b. It is thought that this combination defines a continuum of NK cell maturation with the CD27+CD11b– being the most immature and the CD27–CD11b+ being the most mature. We examined the distribution of NK cell subsets as defined by these two markers in the lungs, spleen and liver of WT B6 mice.

As expected, the liver had the highest concentration of the immature subset while the lung had the highest concentration of the mature subset [[7], [8], [10], [12], [13] and data not shown].

**Figure 5:**
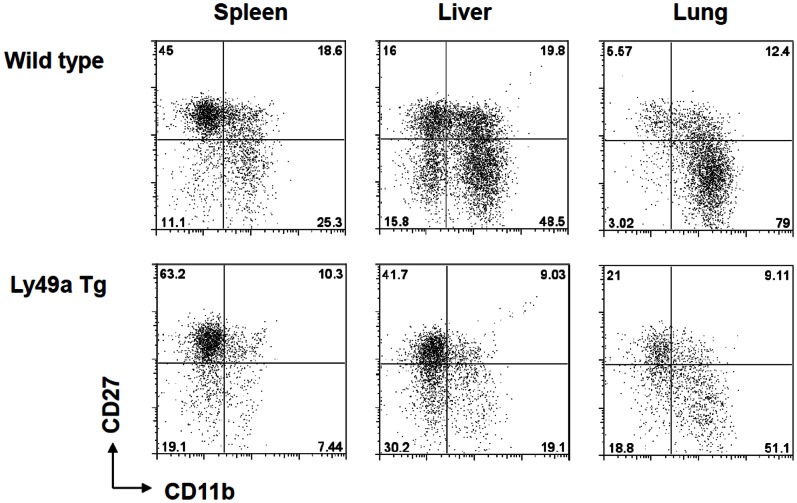
Organ-specific NK cell subset distribution in WT and Ly49A transgenic mice. Mononuclear cells were isolated from the spleens, livers and lungs of wild-type (upper row) and Ly49A transgenic mice (lower row) and analyzed by flow cytometry. Software gates were set to identify NK cells (NK1.1+ CD3–), which were then examined for the expression of the maturation markers CD27 and CD11b.

As mentioned above, the Ly49A transgenic mice have been reported to have a block in NK cell maturation that results in decreased mature NK cells in peripheral lymphoid organs [17], [18]. We confirmed this finding and also examined the hepatic NK cell compartment in the Ly49A transgenic strain. Surprisingly, NK cells were abundant in the liver but appeared to be skewed toward an immature phenotype to a much greater degree than the hepatic cells of WT mice [8], [19]. As shown in Figure 5, Ly49At mice had a skewed concentration of CD27+CD11b– NK cells in all three organs examined. These findings, coupled with those in Figure 4, suggested that immature NK cell subsets play a significant protective role in the liver but not the lung while a mature NK cell subset has the opposite differential benefit.

### Adoptive Transfer of Hepatic NK Cell Subsets into γc RAG-2−/− Mice

**Figure 6:**
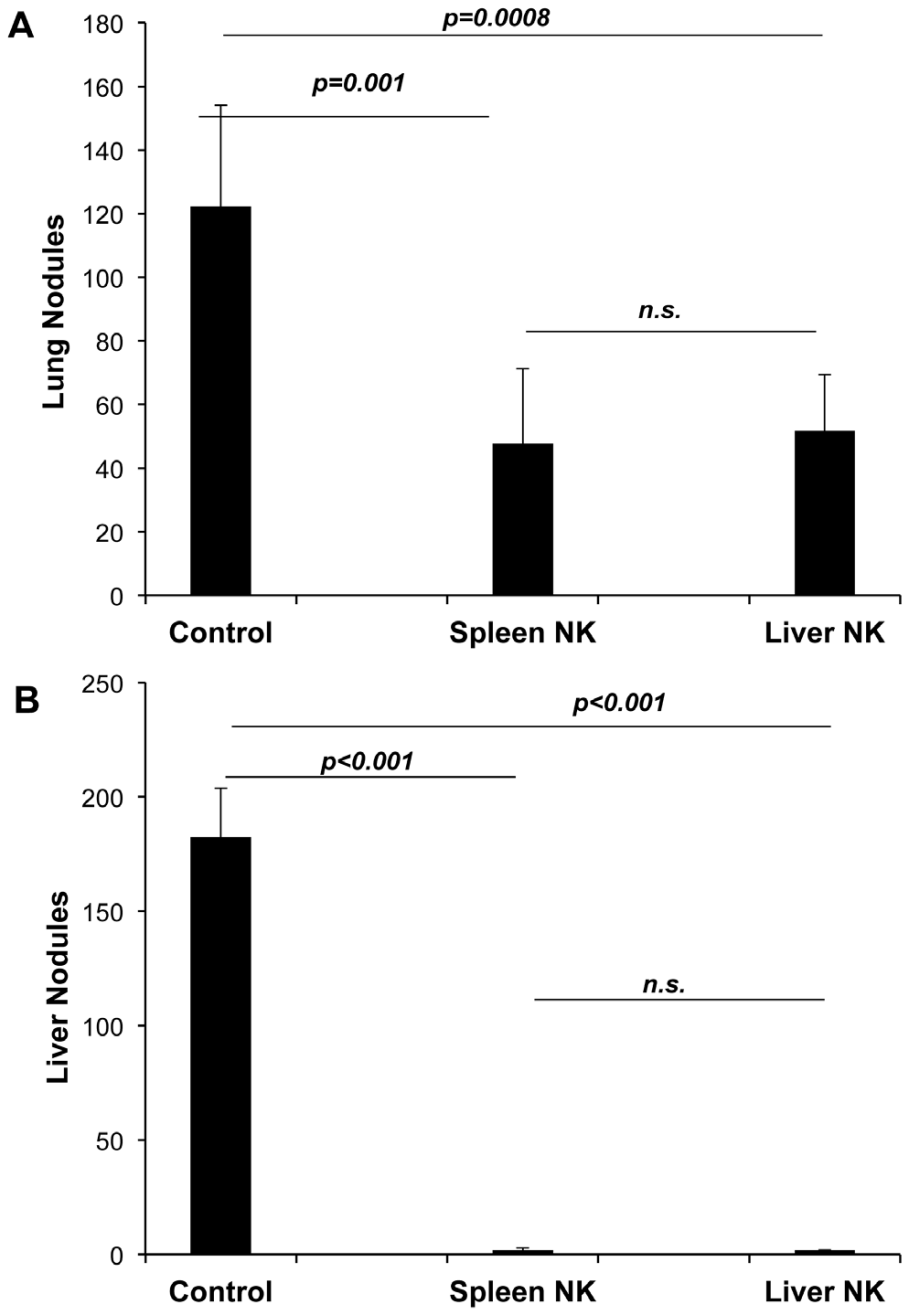
Adoptive transfer of NK cells protects NK cell deficient mice from tumor establishment. γc/RAG2 mice were injected i.v. with 2×10^5^ sort-purified NK cells from the spleens or livers of wild-type C57BL/6 mice. Two weeks later, recipients were inoculated intravenously with 2×10^5^ melanoma cells. Fourteen days later lungs and livers were excised and tumors enumerated. Six recipients were used per group.

In order to confirm that the CD27+CD11b– subset is indeed the protective subset, we performed adoptive transfer experiments. Although we were unable to purify sufficient numbers of pulmonary NK cells, we compared the protection mediated by purified hepatic and splenic NK cells. For these experiments, the recipient mice were γc RAG2−/− mice. Transfer of purified hepatic or splenic NK cells decreased the number of pulmonary tumor nodules but had a greater effect on eliminating the establishment of hepatic metastasis (Figure 6).

**Figure 7:**
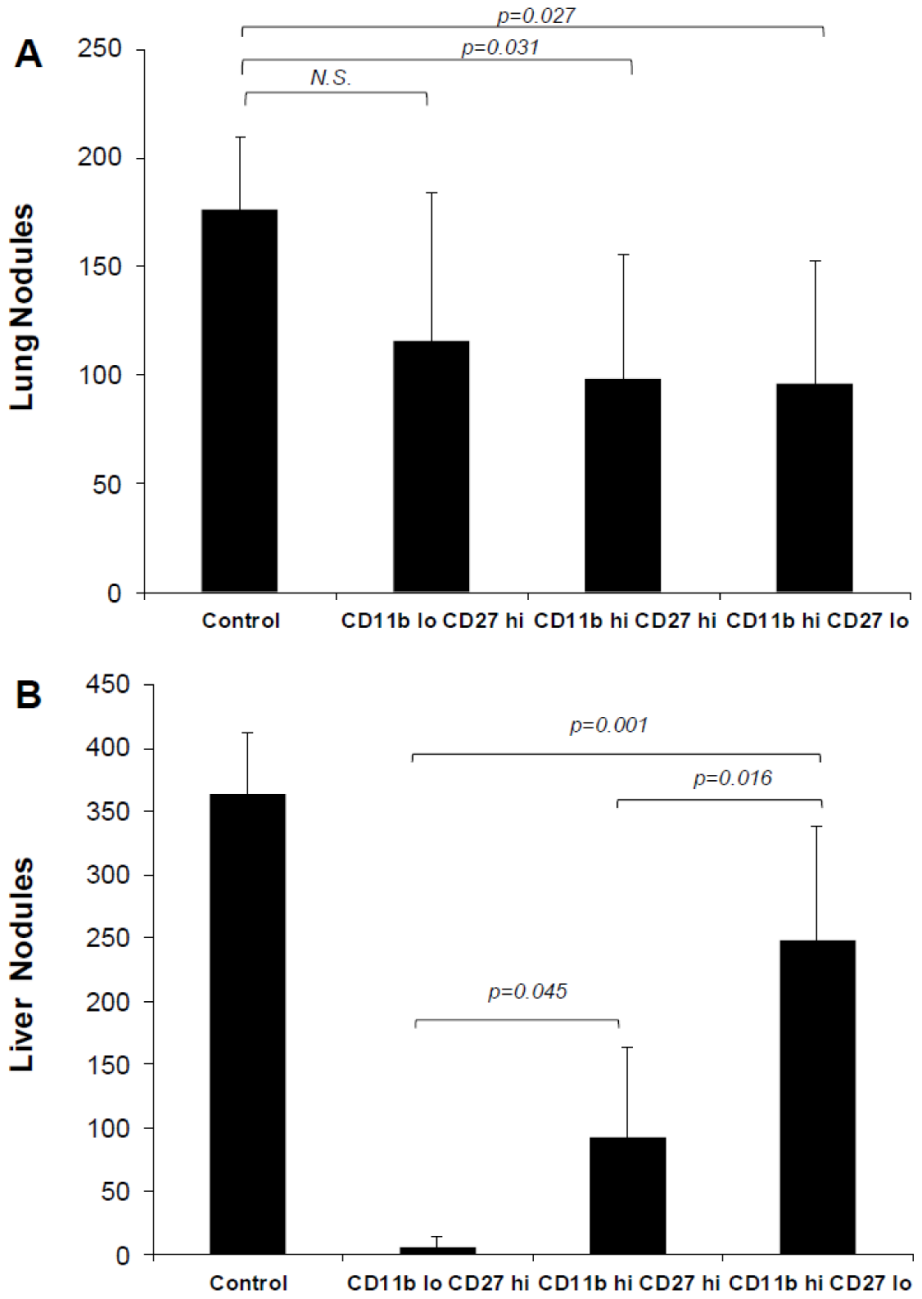
Adoptive transfer of liver NK cell subsets confers variable levels of protection against liver tumor establishment. γc/RAG 2 KO mice were injected i.v. with 5×10^4^ sort-purified NK cells of the indicated subset phenotypes from the livers of wild-type C57BL/6 mice. Recipients were then inoculated intravenously with 3×10^5^ melanoma cells. Fourteen days later lungs and livers were excised and tumors enumerated. Five recipients were used per group.

We next purified hepatic NK cells into three subsets as defined by their co-expression of CD27 and CD11b. We obtained minimal numbers of CD27-CD11b– cells and were not able to examine this subset. However, each of the remaining subsets was transferred intravenously into γc RAG2−/− mice followed by challenge with B16 melanoma. As shown in Figure 7A, there was a hierarchy of protection in the lungs with the most immature subset being non-protective while the two more mature subsets reduced the tumor load significantly albeit not totally. In contrast, the effect of the various subsets on hepatic metastasis was the opposite in the that the most immature subset was most protective; there was a progressively diminishing protective effect which was inversely proportional to the degree of NK cell subset maturity (Figure 7B).

### Differential Cytotoxic Activity of Hepatic NK Cell Subsets

**Figure 8:**
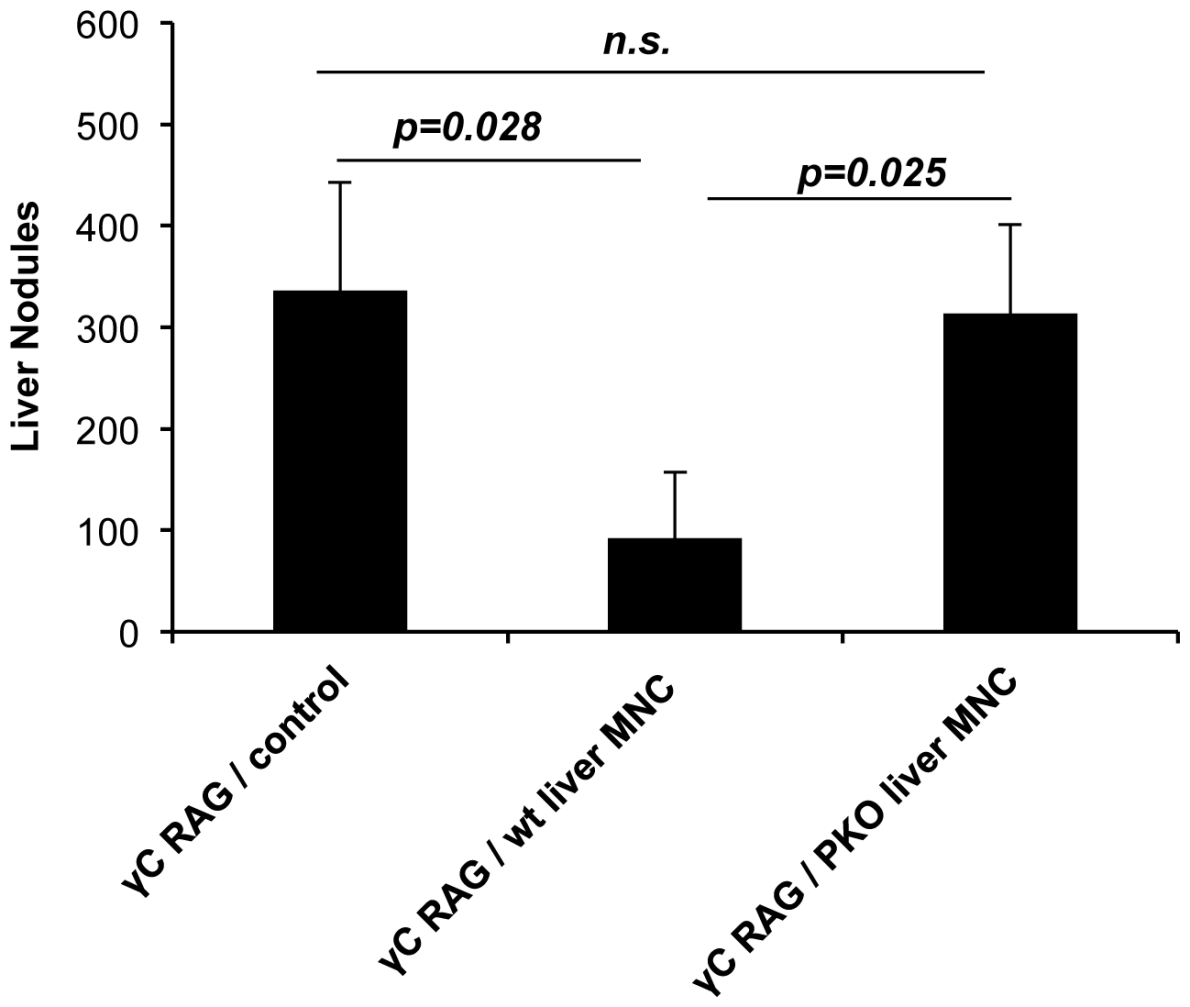
Liver mononuclear cells from perforin deficient mice do not confer protection from liver tumor colonization in NK cell deficient recipients. γc/RAG 2 KO mice or wild type control mice were injected intravenously with 1×10^6^ liver MNC from wild type or perforin deficient C57BL/6 mice. Four days later, recipients were inoculated intravenously with 2×10^5^ B16F1 melanoma cells. Fourteen days later livers were excised and tumors nodules were enumerated.

The data presented above are consistent with hepatic NK cell-mediated protection against B16 metastasis in a perforin-dependent manner. In order to confirm this finding we first sought to determine as to whether adoptive transfer of perforin-deficient NK cells would fail to prevent liver metastasis. This, indeed, was the case as shown in Figure 8. Adoptive transfer of hepatic NK cells from WT mice was efficient at inhibiting liver metastasis but transfer of hepatic NK cells from perforin knockout mice failed to confer significant protection confirming that liver protection is probably mediated by a cytotoxic property of hepatic NK cells.

**Figure 9:**
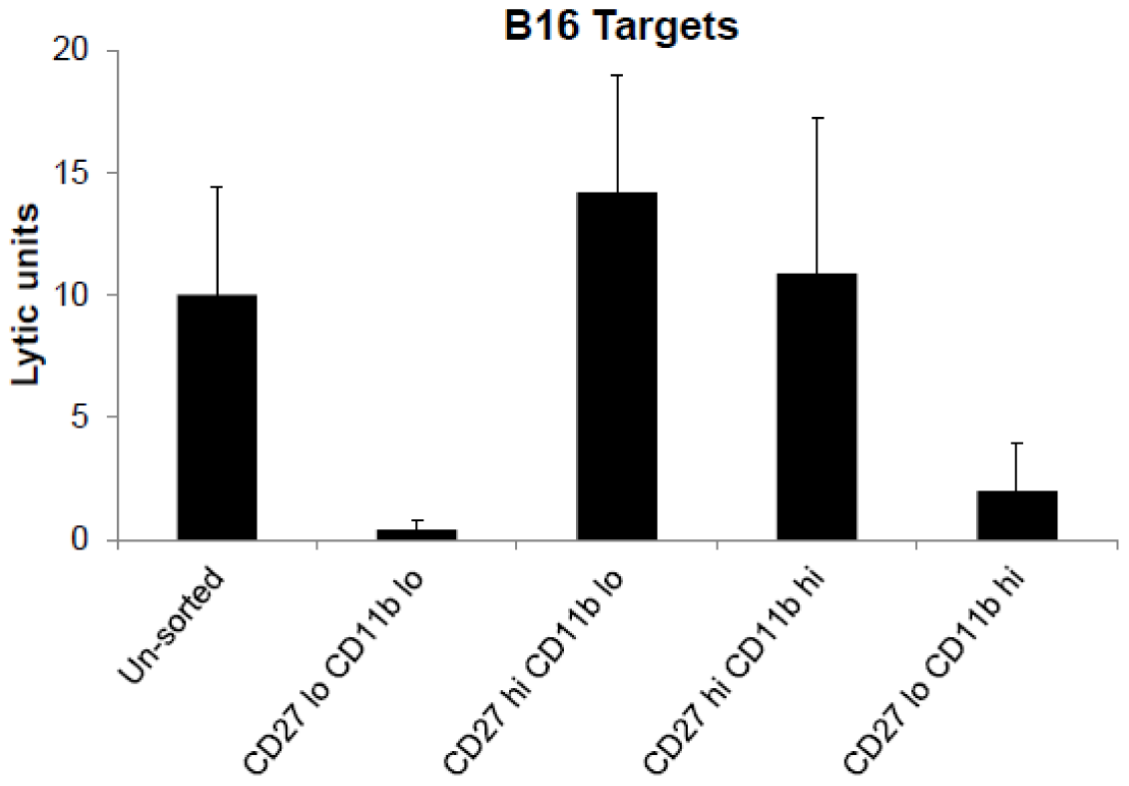
Lysis of B16 melanoma by hepatic NK cell subsets. B6 mice were given CpG i.p, their livers were harvested 48 hrs later, a mononuclear cell preparation obtained and labelled with CD5, NK1.1, CD27 and CD11b. Software gates were set on NK cells and the resulting four subsets of CD27 and CD11b dual label were sorted and used in a 4 hr ^51^Cr-release assay against B16 melanoma targets. Lytic units were calculated with one lytic unit defined as the number of effector cells needed to effect 30% specific lysis. The values on the X-axis are lytic units per 1×10^6^ effector cells. The lytic potential of the CD27^hi^/CD11b^lo^ subset was statistically significant when compared to the CD27^lo^CD11b^lo^ (p = 0.03) and the CD27^lo^CD11b^hi^ (p = 0.028) but not when compared to the CD27^h^iCD11b^hi^ subset.

We sought to demonstrate whether hepatic NK subsets could kill B16 melanoma *in vitro*. In several approaches and experiments not shown, we were not able to demonstrate such killing. One approach that proved useful was to give CpG ODN intraperitoneally to wild type mice and harvesting their spleens, livers and lungs two days later. These cells were then used as effectors in a 4-hour ^51^Cr release assay against YAC-1 (the classical murine NK cell target) or B16 melanoma [6], [20]. As shown in Table 1, CpG was able to induce hepatic effectors capable of killing B16. It appeared possible that CpG might change the balance of the NK subset distribution in the liver. We examined this possibility directly. CpG was administered *in vivo* as above; two days later hepatic NK cells were prepared and were sorted by virtue of their co-expression of CD27 and CD11b (each subset was >95% purity) and then used as effectors in a ^51^Cr-cytotoxicity assay. As shown in Figure 9, the CD27^hi^ CD11b^lo^ was the most efficient cytotoxic subset with CD27^hi^CD11b^hi^ being less active while the other two subsets mediated little or no cytotoxicity. These data demonstrate concordance between the hepatic subset that is most efficient at preventing liver metastasis and the hepatic subset that is most efficient at killing B16 target cells *in vitro*.

**Table 1:** Induction of organ-specific cytotoxicity against B16.

	YAC-1 TARGETS			B16 TARGETS		
	*Spleen*	*Liver*	*Lung*	*Spleen*	*Liver*	*Lung*
**In vivo stimulus**						
CONTROL	**5.69**	**1.19**	**8.65**	**0.00**	**0.00**	**0.00**
CpG	**21.7**	**83.58**	**21.12**	**6.85**	**62.93**	**6.84**

B6 mice were given 100 µg CpG or control ODN intra-peritoneally. Forty-eight hrs. later, the spleens, livers and lungs were obtained and mononuclear cells prepared and used as effectors in a 4 hr-^51^Cr-release assay against YAC-1 or B16 melanoma targets. These data represent % specific lysis at a 50∶1 or a 100∶1 effector: target ratio against YAC-1 and B16, respectively. This experiment is representative of three similar experiments.

## Discussion

The experiments presented in this report were undertaken to test the hypothesis that organ-specific NK cell subsets play a major role in determining permissiveness of organ-specific metastasis using a murine B16 melanoma model. Our data clearly indicate that NK cells are essential in preventing liver metastasis of the B16 melanoma. The data presented in this report also indicate that immature NK cell subsets control tumor metastasis by a perforin-dependent rather than an IFN-γ/IL-12-dependent mechanism. Moreover, the data in this report establish a hierarchy of protection of NK cell subsets along the maturation pathway with the mature subsets exerting some protection in the lungs while the immature subsets were quite efficient at inhibiting hepatic metastasis.

It is thought that both human and murine NK cells go through a continuum of maturation identified by the differential acquisition of CD56 and CD16 (in humans) and, among other markers, the differential acquisition of CD27 and CD11b. It is also thought that the immature NK cell subsets are the predominant cytokine secretors while the mature NK cell subsets are mostly cytotoxic [21]. Our data examining the organ-specific distribution of mature and immature NK cells are in agreement with the findings of others previously reported in the literature [7], [8], [10], [11]. Our data also clearly indicate that an immature NK cell subset in the liver is central in preventing B16 melanoma hepatic metastasis and that it does so by a cytotoxic mechanism rather than by cytokine (IFN-γ and IL-12) secretion. One can argue that the combination of CD27 and CD11b may not be discriminatory enough to identify the various NK cell subsets. While this is a germane argument, our experiments with Ly49A transgenic mice support the hypothesis that an immature NK cell subset is essential. Ly49A transgenic mice have clearly been shown to have a block in the NK cell maturation pathway at an early developmental stage. We have confirmed this by staining for the CD27 and CD11b markers (Fig. 5). The data in Figure 3C clearly indicate that these mice have increased pulmonary metastasis but have no hepatic metastasis thus supporting the idea the immature NK cells are protective against hepatic B16 melanoma. Also, depletion of all NK cell subsets in these mice had no effect on the pulmonary tumor load while allowing hepatic tumor establishment again suggesting that the mature NK cells are more important in the lung while the hepatic NK cells (even when blocked at an early maturation phase) are more protective in the liver.

The experiments using perforin knockout mice, adoptive transfer of perforin-deficient hepatic NK cells and the demonstration of direct cytotoxicity of B16 melanoma by the immature NK cell subsets clearly indicate that the CD27+ CD11b– immature subset in the liver contains cytotoxic cells and that it is their cytotoxicity that is protective against liver metastasis.

The data presented in this report also raise an important question about the role of the various NK cell subsets in the development of pulmonary metastasis. A trivial explanation is that NK cells are not important and that the reason lung nodules are readily apparent while liver tumor is not is because of mechanical trapping of the tumor in the lung since the tumor is given intravenously. We do not believe this to be the case for several reasons. In data not shown, we used progressively increasing numbers of tumor cells for i.v. injection; no hepatic tumor was seen even when the lungs had a massive tumor load. Our experiments with NK depletion and with γc RAG −/− mice clearly indicate that in the absence of NK cells, the tumor easily goes to the liver hence mechanical trapping cannot be playing a significant role. Moreover, NK-deficient mice and NK-depleted mice (as well as perforin −/−) mice consistently and reproducibly exhibited a highly significant increase in the pulmonary tumor load as compared to their appropriate controls. Perhaps the most convincing argument comes from our findings with Ly49At mice. These mice consistently had a much larger tumor load in the lungs as compared to WT mice but still did not have hepatic metastasis arguing against the possibility that liver metastasis is a “spill-over” from the lungs. Moreover, NK cell depletion Ly49At mice did not result in an increased pulmonary tumor load but clearly resulted in hepatic metastasis that was not present in the NK-replete Ly49At mice. Nevertheless, in order to approximate the human situation, we are currently examining differential organ metastasis using an orthoptic tumor model.

The observation that NK depletion (in mutant mice or by injection of anti-NK1.1 antibody) increased the pulmonary tumor load suggests that pulmonary NK cells are able to limit but not abolish pulmonary tumor establishment. Mature NK cells tend to dominate in the lung as opposed to the liver. It is possible that the few immature NK cells (CD27+ CD11b–) in the lungs play a role in limiting pulmonary tumor but are not present in adequate numbers to prevent the tumor completely. These findings argue, however, that the mature NK cell subsets in the lung are not sufficient to control the local tumor growth. This may be due to low numbers, due to their lack of cytotoxicity against B16 or due to the possibility that local factors inhibit such cytotoxicity.

Adoptive transfer of hepatic NK cell subsets, while clearly protective against hepatic metastasis, was not as effective against pulmonary tumor. This may be due to the possibility that the immature hepatic NK cell subset homes directly to the liver. Another explanation is that the pulmonary microenvironment induces rapid maturation of the immature NK subset. In data not shown, we found that adoptive transfer of Ly49A transgenic hepatocytes did not reduce pulmonary tumor load thus arguing against rapid maturation and lending credence to the possibility of homing to the liver. In an elegant study, Chiossone et al [22] found that each of the four subsets defined by CD27/CD11b has a uni-directional differentiation pathway. Thus cells that are CD27−/CD11b– gave rise to the other three subsets upon adoptive transfer. CD27+/CD11b– cells gave rise to the other two subsets and so on with CD27−/CD11b+ cells being unable to give rise to any of the other subsets. Therefore, our findings in Figure 7 showing that the CD11b ^lo^/CD27^hi^ adoptively transferred subset being protective in the liver but not the lungs may suggest that these immature NK cells have a preferential homing to the liver. A third possibility is that other factors, present in the liver, but not in the lungs are needed for cooperation with the immature NK subset. Alternatively, hepatic NK cells might be dependent on interaction with antigen presenting cells that are unique to the liver (such as Kupffer cells) [23]. Consistent with this hypothesis, we have previously shown that CpG ODN do not activate NK cells directly but require dendritic cells to exhibit their NK-boosting activity [6], [20], [24], [25].

An obvious question is whether these findings are specific to this melanoma model or whether they apply to other tumor models. We are currently examining this issue even though we believe that it would be naïve to expect all tumors to have the same immune regulatory mechanisms. If that were the case, then one would not expect the clearly established fact that each solid tumor has its own “permissive” organ. Nevertheless, the approach outlined in this report, when applied to other tumors and examining innate and adaptive lymphocytes subsets, may prove useful in determining the unique permissive immunological milieu for each tumor.

Another issue raised by these findings is whether this is specific for mice or whether it has direct clinical application. While identical experiments have not been performed in human melanoma, there are several reports suggesting a correlation between decreased cytotoxic NK cells and poor prognosis in melanoma patients. Hersey et al showed that patients with familial melanoma had low NK cell cytotoxic activity [26]. Jovic *et al* [27] reported impaired perforin-dependent NK cell cytotoxicity in patients with metastatic melanoma. More recently, Holtan *et al* [28] reported a shift in NK cell subsets away from the cytotoxic subset and toward the IFN-γ-secreting subset in the peripheral blood of patients with metastatic melanoma. Clearly, therefore, there is strong evidence indicating that NK cell homeostasis plays a significant role in the control of primary and metastatic melanoma in patients.

In summary, our experiments clearly establish that organ-specific NK cell subsets play a pivotal role in determining organ-specific establishment of B16 melanoma in a murine model. Moreover, NK cells effect their near total protection of the liver and their significant but minimal protection of the lungs by a perforin-dependent mechanism. We also established an organ-specific hierarchy of protection with the mature NK cells being more protective in the lung while the immature NK cell subsets were more protective in the liver.

## Materials and Methods

### Mice

Male C57BL/6 mice aged 6–8 weeks were purchased from the National Cancer Institute (Frederick, MD) and housed in the Animal Resource Facility at the Iowa City VAMC. B6.CB17-*Prkdc^scid^*/SzJ (SCID) and CByJ.B6-*Prf1^tm1Sdz^*/J (perforin KO) were purchased from the Jackson Laboratory (Bar Harbor, ME) and housed in the Animal Resource Facility at the Iowa City VAMC. B10;B6-*Rag2^tm1Fwa^ II2rg^tm1Wjl^* (γc/RAG-2 double-KO) were purchased from Taconic Farms (Hudson, NY) and housed in the Animal Resource Facility at the Iowa City VAMC. IL-12/IFN-γ double-KO mice (obtained from Dr. Joel Kline, University of Iowa, Iowa City IA) have been previously described [29] and were bred in the barrier facilities at the University of Iowa. Ly49A transgenic mice (obtained from Dr. Wayne Yokoyama, Washington University, St. Louis, MO) have been previously described [17], [18] and were bred in the barrier facilities at the University of Iowa. All procedures were performed in accordance with the guidelines of the University of Iowa and the VA Medical Center Animal Care and Use Committees.

### Cell Lines and Tumor Inoculation

The B16.F1 melanoma cell line (provided by Dr. Isaiah Fidler, MD Anderson Cancer Center, Houston, TX) was maintained in DMEM supplemented with 5% heat-inactivated fetal calf serum, L-glutamine, HEPES and non-essential amino acids. In order to prevent antigenic drift, a fresh vial of the tumor was thawed and used every three months. Eleven to fourteen days after injection, mice were euthanized and livers and lungs were excised, fixed with Bouin’s fixative (Polysciences, Warrington, PA) and examined under magnification for the enumeration of tumor nodules. This time frame was selected because the mice were still healthy and because the lung and liver nodules were distinct and thus easy to count. Organ weights were also used and were consistent with nodule enumeration and are thus not presented. For the *in vivo* depletion of NK cells, mice were injected intraperitoneally with 0.2 milligrams of PK136 (BioXcell, West Lebanon, NH) on day −2 and day 0 relative to tumor inoculation. Control mice were injected with mouse IgG (Jackson Immunoresearch, West Grove, PA) in the same manner. We have previously shown that this results in prolonged and near total depletion of NK cells *in vivo* [6], [30]. When examining each manipulation or mutant mouse, the experimental mice were always run with the control mice as a single cohort. This is because the exact number of tumor nodules varied from one control cohort to another depending on the exact growth phase of the inoculated tumore. Such variability was minimized by doing all mice groups for a given experimental point simultaneously.

Unless otherwise specified, each experiment was repeated a minimum of three times.

CpG oligodeoxnucleotides (ODN) were used in some experiments. CpG ODN #1826 (Sigma Life Sciences-Custom Products, The Woodlands, TX) was used and had the following sequence: 5′-TCCATGACGTTCCTGACGTT-3′.

### Isolation of Hepatic and Splenic Mononuclear Cells

Mononuclear cells were isolated from liver and spleen as previously described [31]. Livers were minced and pressed through 100 micron cell strainers (BD Biosciences, Franklin Lakes, NJ), washed and resuspended in 40% Percoll (Sigma Life Science, St. Louis, MO) prior to centrifugation at 800×*g* for 20 minutes. The resultant pellet was treated with 0.83% ammonium chloride to lyse red blood cells. Spleens were homogenized with the frosted ends of microscope slides (Surgipath, Richmond, IL). Red blood cells were lysed by treatment with 0.83% ammonium chloride.

### Antibodies, Flow Cytometry and Adoptive Transfers

Surface staining for flow cytometric analysis was performed as previously described [31]. PK136 (αNK1.1) PE or APC, 17A2 (αCD3) eflour450, 53-7.3 (αCD5) FITC, M1/70 (αCD11b) alexa 488 or PE and LG.7F9 (αCD27) PE or PerCP-eflour 710 were purchased from eBioscience (San Diego, CA). Samples were analyzed on a BD LSRII flow cytometer (BDBiosciences, San Jose, CA). NK cell subset populations were sorted using a BD ARIA II flow cytometer (BDBiosciences, San Jose, CA) at the University of Iowa Flow Cytometry Facility. Sorted populations were thoroughly washed and resuspended in PBS prior to intravenous injection into recipient mice. Sorted populations were used only if post-sort analysis demonstrated >95% purity.

### ^51^Chromium Release Assay

Cytotoxic activity was assessed using a standard 4-hour ^51^Cr release assay directed against YAC-1 targets (ATCC, Manassas, VA) or B16.F1 targets as previously described [6], [16]. For calculation of lytic units, one lytic unit was defined as a quantity of cells sufficient to effect 30% lysis of the targets. The data presented as lytic units indicate the amount of lytic unit per 1×10^6^ effector cells.
